# Limitations of current high-throughput sequencing technologies lead to biased expression estimates of endogenous retroviral elements

**DOI:** 10.1093/nargab/lqae081

**Published:** 2024-07-09

**Authors:** Konstantina Kitsou, Aris Katzourakis, Gkikas Magiorkinis

**Affiliations:** Department of Hygiene, Epidemiology and Medical Statistics, National and Kapodistrian University of Athens, Athens 11527, Greece; Department of Zoology, University of Oxford, Oxford OX1 4BH, UK; Department of Hygiene, Epidemiology and Medical Statistics, National and Kapodistrian University of Athens, Athens 11527, Greece

## Abstract

Human endogenous retroviruses (HERVs), the remnants of ancient germline retroviral integrations, comprise almost 8% of the human genome. The elucidation of their biological roles is hampered by our inability to link HERV mRNA and protein production with specific HERV loci. To solve the riddle of the integration-specific RNA expression of HERVs, several bioinformatics approaches have been proposed; however, no single process seems to yield optimal results due to the repetitiveness of HERV integrations. The performance of existing data-bioinformatics pipelines has been evaluated against real world datasets whose true expression profile is unknown, thus the accuracy of widely-used approaches remains unclear. Here, we simulated mRNA production from specific HERV integrations to evaluate second and third generation sequencing technologies along with widely used bioinformatic approaches to estimate the accuracy in describing integration-specific expression. We demonstrate that, while a HERV-family approach offers accurate results, per-integration analyses of HERV expression suffer from substantial expression bias, which is only partially mitigated by algorithms developed for calculating the per-integration HERV expression, and is more pronounced in recent integrations. Hence, this bias could erroneously result into biologically meaningful inferences. Finally, we demonstrate the merits of accurate long-read high-throughput sequencing technologies in the resolution of per-locus HERV expression.

## Graphical Abstract

**Graphical Abstract:**
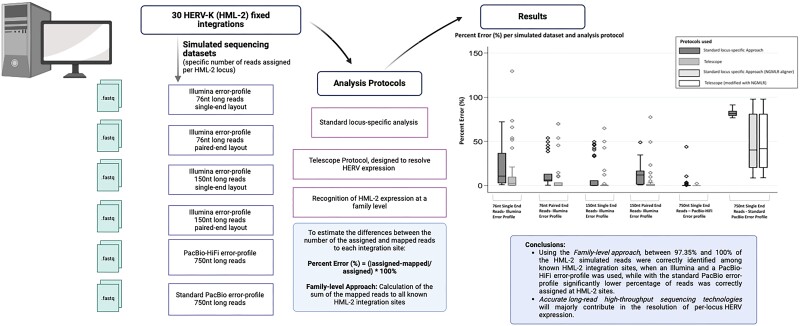

## Introduction

The interplay between viruses and their mammalian hosts is significant for both the preservation of health and the development of disease (1). Integral parts of the human virome are human endogenous retroviruses (HERVs), the remnants of ancient retroviral infections, which currently comprise almost 8% of the human genome (1,2). The majority of the HERV integrations have become inactivated, either with the accumulation of multiple mutations, and recombination events between their long terminal repeats (LTRs), leading to the creation of solo-LTRs (3), or with strict transcriptomic control of HERV expression (4).

The only family known to have proliferated in the human ancestors’ germline after the human-chimp split is HERV-K (HML-2) until at least 1 MY ago (5), with several of these integrations demonstrating functional open reading frames that encode viral proteins, and its expression has been detected in various human tissues in health and disease (6). Interestingly, the expression of HERVs has been associated with a plethora of modern human diseases including cancer and autoimmune conditions either through the transcription of nucleic acids or virally encoded proteins (7). Establishing causality between HERV expression and disease manifestations in humans is challenging, however novel advances allow us to approach this in ways that were not previously considered possible. Long-read high-throughput sequencing data production is very promising for the detection and quantification of HERV expression (8,9) and multiple bioinformatics approaches and software packages have been used (10–12). No single approach, however, has been conclusively proven to yield optimal results. The development of the most appropriate strategies for the detection of HERV expression remains a major challenge in the study of their role in human disease, as the high error rates of the widely available high-throughput sequencing technologies (13) and the highly repetitive nature of these elements are important obstacles for the production of unambiguous results.

In this simulation analysis, we aim to compare the relative limitations of locus-specific and family-wide approaches in the determination of HERV element expression in high-throughput sequencing data. We show that a locus-specific determination of HERV element expression is highly-likely to erroneously show that some integrations are being significantly upregulated or downregulated, even though at the same time the family-wide approach offers an accurate and unbiased characterization of HERV expression. We also show that this locus-specific bias can only be partially mitigated by more advanced algorithms and is more intense in younger HERV integrations suggesting that the locus-specific bias may lead to unsupported biologically-meaningful interpretations. Finally, we show that an unbiased locus-specific analysis can take place when long-read high-throughput sequencing technologies achieve an error rate comparable to the current small-read approaches.

## Materials and methods

This is a simulation-based analysis. HML-2 has expanded in the genome of our ancestors for at least 20 million years (6,14,15), and the human genome demonstrates multiple integrations whose age span variably (6).

We randomly chose 30 HML-2 integration sites, ten from three distinct groups based on their integration age, as described by Subramanian *et al.* (6). To overcome the estimated range of age of some of the integration sites, we used the mean of the minimum and maximum estimated age, as a surrogate of the actual age of the elements in question. Similarly, in case of elements with an estimated integration age of <2 MY, we used 1.9 MY as an arithmetic proxy for this analysis and for graphical presentation. ‘Young’ elements included had an estimated age less than 2 MY up to 9.38 MY, ‘intermediate age’ elements with an estimated integration time from 9.83 MY to 24.24 MY, and ‘old’ HML-2 integrations, with estimated age from 20.03 up to 53.79 MY. The genomic coordinates, the age, whether they are solo-LTRs or proviral elements, and the group of the categorization used for this study of the integration sites can be found in the Supplementary Table S1.

After extracting the sequences corresponding to the coordinates of these elements from the Homo sapiens (human) genome assembly GRCh37 (hg19), using the Bedtools getfasta command with default settings (16), we used the BBMap randomreads.sh command (17), to simulate .fastq files of commonly used sequencing technologies. More specifically, using the Illumina error profile, we produced the following short- and medium-read .fastq files: 76nt long reads single-end layout, 76nt long reads paired-end layout, 150nt long reads single-end layout, and 150nt long reads paired-end layout. Furthermore, using the standard PacBio error profile, we simulated .fastq data of 750nt long reads single-end layout.

To examine the effect of highly accurate long reads in the detection of repetitive elements, we also aimed to simulate the PacBio-HiFi error profile, which is characterized by an estimated accuracy of 99.8% (18–21). To our knowledge, no currently used sequencing simulator offers the potential of simulating long-reads with an error profile as such. Thus, we used an Illumina-like error profile to simulate data of 750nt long reads single-end layout, to resemble this technology.

To be able to compare the transcription levels among datasets with different read lengths, we adjusted the number of raw reads corresponding to each integration site to obtain the same coverage. For this reason, we used an estimated length of 1000nt for the solo-LTRs of HML-2, and 10 000nt for the HML-2 proviruses included in this analysis.

### Family-level approach

We used Bowtie2 with default settings for single-end and paired-end data, accordingly, for the alignment to hg19 human genome assembly to produce a .sam file (22). We processed the Bowtie2 output files, using Samtools view, with the use of the -b option to produce a .bam file output and subsequently Samtools sort, to write a sorted .bam file. Finally we used the Samtools index command with default settings to index the coordinate-sorted .bam files for further processing (23). To retrieve the number of raw reads corresponding to each of the HML-2 loci for every read-sequencing technology, in each of the datasets, we used the Bedtools multicov command with default settings (16). To be able to perform this step we used a .bed file of the coordinates of the HML-2 integration sites described by Subramanian et al, as the HML-2 annotation (6).

To quantify the transcription of HML-2 in our simulated files, we wrote the Bedtools multicov output in a text file for further processing. Finally, we calculated the sum of the reads aligned to all the HML-2 integration sites, to retrieve all reads that were aligned to HML-2 sites, thus evaluating the transcription at a family level (6).

### Telescope implementation

As a comparator, we used the Telescope software, which was designed for the recognition of the expression of transposable elements at an integration locus level. Telescope uses a Bayesian statistical model to reassign uncertainly mapped fragments to their most probable source (11).

We conducted the alignment of our simulated .fastq files again, this time with alignment options set to perform a sensitive local alignment search as described by Bendall et al (–very-sensitive-local, -k 100, –score-min L,0,1.6) (11). For the implementation of the Telescope software, we converted the HML-2 annotation we used to the .gtf format with the use of bedtoGenePred and genePredToGtf commands from the UCSC tools (33). We set the Telescope options as described by Bendall et al (–max_iter 200, –theta_prior 200000) (11). We used the ‘final counts’ column from the Telescope output for the raw mapped reads per integration site simulated.

### Standard PacBio data mapping

Regarding the standard PacBio simulated dataset, bearing in mind that the usually used long-read sequencing technologies come with certain limitations regarding their error profiles and efforts have been made to overcome these issues by the development of specialized bioinformatics approaches, we have also conducted an analysis of this dataset with the NGMLR aligner for long-read sequencing data with default setting (24). The NGMLR output file was processed with the Samtools view, sort and index commands with default settings (23), and Bedtools multicov command was used as indicated above (16). We extracted the per locus transcripts for the simulated loci, and we also calculated the sum of the total transcripts mapped to all the HML-2 loci in the used annotation.

Also, we used the Telescope software with the indicated settings as above, this time with the NGMLR output file, to examine whether specialized approaches as such increase the accuracy in long-read sequencing data analysis. We used the ‘final counts’ column from the Telescope output for the raw mapped reads per integration site simulated.

### Methods comparison

To be able to objectively estimate any occurring differences between the number of the assigned reads to each integration site in our simulated datasets and the number of the reads that were allocated to each of the HML-2 integration sites during the analysis, with each of the approaches described above, we used the percent error (%), calculated as:


\begin{eqnarray*} {\mathrm{Percent\ Error\ }}( {\mathrm{\% }} ) = ( {| {{\mathrm{assigned}} - {\mathrm{mapped}}} |/{\mathrm{assigned}}} ){\mathrm{\, *\, }}100{\mathrm{\% }} \end{eqnarray*}

Furthermore, we calculated the percentage of the assigned reads (as a total) that were detected at a Family level, with each method, by dividing the sum of the raw mapped reads to the sum of the simulated reads in each dataset simulated.

We calculated the fold change in the detected reads compared to the prespecified in the simulated datasets, as the quotient of the mapped to the assigned reads in each locus. We considered as falsely up- and downregulated loci, those for which the fold change is either >1.1 or <0.9, respectively.

The number of the erroneously upregulated and downregulated loci (i.e. loci where higher or lower -respectively- number of reads were mapped during the bioinformatics analysis than the originally assigned during the simulation of the datasets) per analysis protocol and its fold change in reference to the assigned reads in each simulated dataset are shown in Table 1.

**Table 1.** Percent Error (%), as a measure of the bias (difference between the assigned and the mapped reads per integration site) in each of the simulated datasets and comparison between the standard locus-specific approach and the use of Telescope

Percent error (%) in each of the simulated datasets and comparison between the two approaches used														
				Falsely upregulated loci [n(%)]		False upregulation fold change^a^ [range]		Falsely downregulated loci [n(%)]		False downregulation fold change^a^ [range]		Percent error (%) [median(range)]		
*Error Profile*	*Length (nt)*	*Aligner*	*Layout*	*Locus-specific Approach*	*Telescope*	*Locus-specific Approach*	*Telescope*	*Locus-specific Approach*	*Telescope*	*Locus-specific Approach*	*Telescope*	*Locus-specific Approach*	*Telescope*	*p-value*
Illumina	76	Bowtie2	single-end	0 (0)	1 (3.33)	-	2.29	15 (50)	6 (20)	0.34–0.88	0.34–0.79	10.58 (0.89–72.13)	2.55 (0.04–129.47)	*<0.001*
Illumina	76	Bowtie2	paired-end	0 (0)	1 (3.33)	-	1.7	8 (26.66)	6 (20)	0.46–0.87	0.30–0.89	6.42 (0.4–53.81)	0.24 (0–69.91)	*0.003*
Illumina	150	Bowtie2	single-end	0 (0)	1 (3.33)	-	1.65	7 (23.33)	4 (13.33)	0.51–0.67	0.50–0.88	0.15 (0–49.43)	0 (0–64.9)	*0.001*
Illumina	150	Bowtie2	paired-end	0 (0)	1 (3.33)	-	1.23	21 (70)	3 (10)	0.51–0.89	0.23–0.86	12 (0.71–48.87)	0 (0–77.41)	*0.001*
PacBio-HiFi	750	Bowtie2	single-end	0 (0)	0 (0)		-	1 (3.33)	0 (0)	0.56	-	0 (0–43.79)	0 (0–2.21)	*0.025*
Standard PacBio	750	Bowtie2	single-end	0 (0)	-	-	-	30 (100)	-	0.09–0.24	-	81.5 (76.63–91.25)	-	-
		NGMLR	single-end	0 (0)	0 (0)	'-	'-	29 (96.66)	29 (96.66)	0.02–0.85	0.02–0.85	42.83 (8.66–97.92)	42.85 (8.75–97.92)	0.999

^a^False up- and downregulation for the loci analyzed is determined as a quotient (mapped/assigned reads) >1.1 or <0.9 respectively, indicating a higher or lower (respectively) number of reads mapped during bioinformatic analysis compared to those assigned during the simulation of the analyzed datasets.

*P*-values italics indicate statistically significant results, Mann–Whitney *U* test.

False modification of expression is considered as a quotient between mapped and assigned reads <0.9 or >1.1.

### Effect of the high-throughput sequencing technology characteristics and the accuracy of the locus-specific integration detection

To evaluate the effect of the read length on the accuracy of the detection of the HERV level transcription in our simulated datasets, we compared the calculated percent error (%) among the singe-end lay-out datasets with different read lengths (short-, medium- and long-read length technologies) and the percent error (%) between the two paired-end lay-out datasets (short- and medium-read length technologies). These comparisons were conducted for both the analysis approaches we used, once for the standard locus-specific protocol and once for the implementation of Telescope and in the case of the standard PacBio simulated dataset, we included the NGMLR alignment analysis in these comparisons.

### Effect of the age of HERV integrations on the efficiency of per locus detection of transcription

To examine the effect of the age of each HML-2 integration on the accuracy of the per locus detection of transcription, we correlated the percent error (%) we previously calculated to the estimated age of the HML-2 elements, that we used in this simulation. We, also compared the occurring Percent Error (%) among ‘young’ elements, ‘intermediate age’ elements, and ‘old’ HML-2 integrations, as described above, for the two examined approaches.

### Analysis of the behaviour of HERV proviral elements

Given the higher number of solo-LTRs included in the simulated datasets, which reflects their abundance in the human genome compared to full-length or near full-length proviruses, we also conducted a sensitivity analysis in order to evaluate whether any estimated bias inferred through this analysis is applicable in the study of the behaviour of HML-2 proviral elements with the use of the studied sequencing technologies. We, thus additionally, simulated .fastq datasets of the HML-2 proviral sequences included in this study (*n* = 12), using the Illumina error profile using 76nt long reads single-end layout, 76nt long reads paired-end layout, 150nt long reads single-end layout, and 150nt long reads paired-end layout. Furthermore, using the standard PacBio and the PacBio-HiFi error profiles, we simulated .fastq data of 750nt long reads single-end layout, and these files were processed as described in the corresponding sessions above and we conducted the aforementioned comparisons among the sequencing layouts regarding their performance on the accuracy on the detection HERV proviral expression and the effect of the age of HERV proviral integration on the accuracy of expression detection.

### Statistical analysis

The median of the percent error (%), and its minimum and maximum values for each of the datasets we simulated and the two analysis approaches we used, is shown in Table 1, since it is not normally distributed. Furthermore, the number of the upregulated and downregulated loci per analysis protocol and its fold change in reference to the assigned reads in each simulated dataset are shown in Table 1.

We conducted the Mann–Whitney *U* test for the comparisons between the calculated percent error (%) that occurred with the standard analysis we describe above and the percent error (%) that occurred when the Telescope protocol was utilized, for each of the simulated datasets (Table 1). To evaluate the effect of the read length on the accuracy of the detection of the HERV level transcription in our simulated datasets, we used Mann–Whitney *U* test to compare the percent error (%) among the singe-end lay-out datasets with different read lengths and to compare the Percent Error (%) among the two paired-end lay-out datasets (short- and medium-read length).

For each of the read-sequencing technologies simulated in this work, Spearman's rank correlation test was conducted to investigate the correlation of the percent error (%) with the estimated age of the loci included in this analysis. We conducted Mann–Whitney *U* test for the comparisons between the percent error (%) among ‘young’ elements, ‘intermediate age’ elements, and ‘old’ HML-2 integrations, as described above, for the two examined approaches.

All tests are two-tailed and *P*< 0.05 was considered as statistically significant. IBM Corp. Released 2015. IBM SPSS Statistics for Windows, Version 23.0. Armonk, NY: IBM Corp. was used for the analyses in this work. StataCorp. 2009. Stata Statistical Software: Release 11. College Station, TX: StataCorp LP and GraphPad Prism version 6.0.0 for Windows, GraphPad Software, Boston, Massachusetts USA, www.graphpad.com were used for the figures in this work.

## Results

### Integration-specific expression analysis results in locus-specific expression bias

Using a standard locus-specific approach, we found 3–100% of the loci included in this simulation to be falsely downregulated in the simulated datasets (Table 1). In the simulated datasets, where an Illumina error profile was used, we found 3–70% of the loci to be falsely downregulated, whilst the expression of all the HML-2 loci was underestimated in the standard PacBio error profile simulated dataset (Table 1).

**Figure 1.**
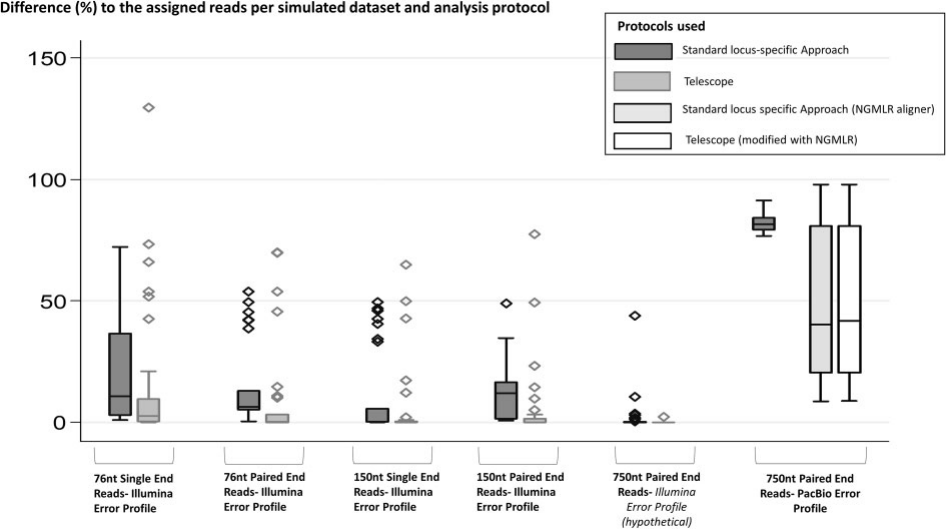
Box-plots of the bias calculated as percent error (%) between the assigned reads and the mapped reads to each integration site (*N* = 30) in the simulated datasets for each of the analysis protocols used. Minimum values, the first quartile, the median, the third quartile and the maximum values are indicated per protocol in the figure. Significantly lower bias occurred in all cases of the Illumina error profile simulated datasets with the use of the Telescope software (76nt single-end Illumina error-profile: *P*< 0.001; 76nt paired-end Illumina error-profile: *P*= 0.003; 150nt single-end Illumina error-profile: *P*= 0.001; 150nt paired-end Illumina error-profile: *P*= 0.001; 750nt PacBio-HiFi error-profile: *p*= 0.025).

Telescope demonstrated lower Percent Error (%) in the Illumina error profile simulated datasets than the standard locus-specific analysis (*P*< 0.05 in all cases) (Figure 1, Table 1). However, locus-specific expression bias could not be mitigated. More specifically, with this approach up to 20% of the simulated loci were found up- or downregulated in small- and medium-read-length Illumina error-profile datasets (Table 1). For example, HML-2 locus chr8:7355397–7364859 was erroneously found 2.29-fold upregulated in the single-end 76nt long read dataset, for which a bias of 129.47% was calculated (Table 1). No falsely downregulated or upregulated loci were found in the PacBio-HiFi dataset. We were not able to use Telescope with its indicated protocol on the standard PacBio profile simulated reads, as local bowtie2 alignment cannot be conducted with long reads with this error profile, thus we only performed a standard locus-specific analysis in this dataset. We also observed that the integration site chr1:13678850–13688242, consistently, led to low percentages of detection of the HML-2 simulated transcripts with the use of the standard locus-specific analysis, with a bias that constituted a high outlier in all the cases (Figure 1). This site demonstrates 100% similarity to an adjacent HML-2 provirus (located at chromosome 1, chr1:13458015–13467406), which can be attributed to a duplication event of the HML-2 provirus after its integration. The transcription of this integration site was correctly detected with the use of the Telescope software in all the cases of short- and medium-length read simulations and we assume that Telescope allocates all the reads, similar to one locus, to one specific site that they appear to correspond regardless of the existence of sequences with high identity percentage to this locus (Supplementary Data).

The use of the NGMLR aligner improved the performance of the standard per-locus approach (*P*< 0.001) compared to the standard approach with the use of Bowtie2, but still most of the loci included in the simulation were found falsely downregulated compared to their assigned transcription (Table 1). We were able to perform the Telescope software analysis on the NGMLR output, and the accuracy of the per-locus detection did not differ with either of the two approaches (*P*= 0.999). During this analysis, the reads assigned to the possibly duplicated locus chr1: chr1:13678850–13688242, were not assigned to a single of the two identical loci, but were rather distributed to both, owing to the high error rate and the low level of identity among the reads simulated using the standard PacBio error profile.

On the other hand, by means of the Family-level approach by summing the total read counts aligned at annotated HML-2 loci in the human genome, in the case of all the Illumina error-profile datasets, we correctly assigned between 97.35% and 100% of the HML-2 simulated reads. Regarding the long-read simulated dataset with a standard PacBio error profile even after calculating the sum of the mapped reads in all the HML-2 integration sites in our annotation we identified only 21.73% of the simulated reads with the use of Bowtie2, while with the NGMLR aligner, at a family-wide level, we were able to assign to HML-2 loci 55.23% of the simulated reads.

### Longer reads lead to higher accuracy of the locus-specific integration detection provided there is a low error rate

Generally, longer reads led to smaller bias between the mapped and the prespecified reads in the simulated datasets (Figure 1).

Using the standard locus-specific approach, regarding single-end layout datasets with an Illumina error-profile, 76nt long reads demonstrated significantly higher bias compared to 150nt long reads (*P*< 0.001) and 150nt long reads demonstrated higher bias compared to the PacBio-HiFi 750nt long reads (*P*< 0.001). However, when we compared the standard PacBio error profile simulated dataset to the 76nt Illumina error-profile single-end reads, the standard PacBio dataset demonstrated higher bias and thus the least accuracy (*P*< 0.001 for both the aligners used with the locus-specific approach). Regarding the paired-end layout datasets, no significant differences were recognized between the 76nt long and the 150 long-read datasets, with the standard approach.

Similarly with the Telescope approach, regarding single-end layout datasets, 76nt long reads demonstrated significantly higher bias compared to 150nt long reads (*P*< 0.001) and 150nt long reads demonstrated higher bias compared to the PacBio-HiFi 750nt long reads (*P*< 0.001). The implementation of the Telescope protocol on the NGMLR aligner output of the standard PacBio simulated dataset led to significantly higher bias compared to Illumina profile 76nt long reads (*P*< 0.001). Using Telescope, paired-end 76nt long reads demonstrated significantly higher bias compared to 150nt long reads.

### The estimated age of HERV integrations inversely correlates with the locus-specific expression bias which can be mitigated with longer-accurate reads

Using a standard locus-specific analysis, we recognized statistically significant correlations between the calculated bias and the estimated age of the elements in the Illumina error profile simulated datasets (*P*< 0.05 in all cases) (Supplementary Figure S1). Regarding the standard PacBio simulated dataset, we found no significant correlation between the estimated bias and the age of the elements by means of the standard locus-specific protocol. On the contrary, we found statistically significant correlations of the bias with the age of the elements in both the standard locus-specific protocol and the Telescope analysis, when we used the NGMLR aligner (*P* < 0.001 in both cases) (Supplementary Figure S1).

With the use of Telescope, we recognized statistically significant correlations between the calculated bias and the estimated age of the elements in small- and medium-length datasets (both single- and paired-end) (*P* < 0.001 in all cases) (Supplementary Figure S1). We found no significant correlation between the bias and the age of the elements for the PacBio-HiFi long-read simulated dataset.

**Figure 2.**
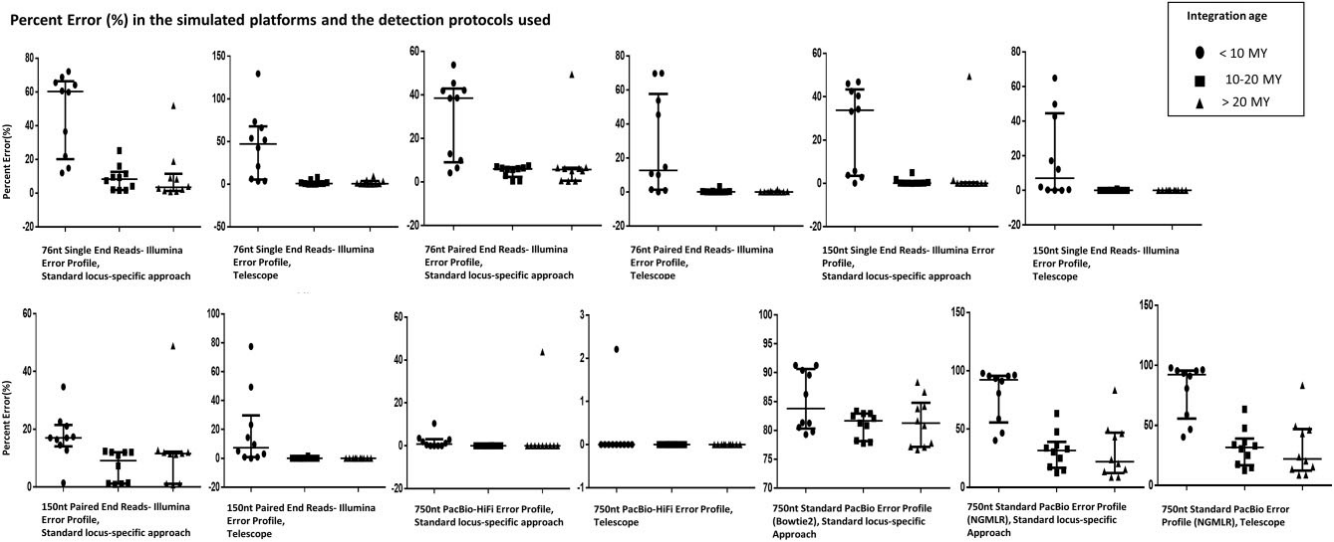
Box-plots of the bias calculated as percent error (%) between the assigned reads and the mapped reads to each integration site, comparison per age group (*N* = 10 integration sites per subgroup). Minimum values, the first quartile, the median, the third quartile and the maximum values are indicated per protocol in the figure. ‘Young’ elements (age < 10MY) demonstrated significantly higher Percent error (%) in datasets: 76nt single-end Illumina error-profile, 76nt paired-end Illumina error-profile, 150nt single-end Illumina error-profile and 150nt paired-end Illumina error-profile compared to elements of ‘intermediate age’ (age = 10–20MY), both with the use of a standard locus-specific approach and telescope (*P*< 0.001–both; *P*= 0.002-both; *P*= 0.001–both; *P*< 0.001–both, respectively), while in the 750nt PacBio-HiFi error-profile dataset we observed significantly higher percent error (%) only with the use of standard locus-specific approach (*P*= 0.035). ‘Young’ elements demonstrated significantly higher bias in datasets: 76nt single-end Illumina error-profile, 76nt paired-end Illumina error-profile, 150nt single-end Illumina error-profile and 150nt paired-end Illumina error-profile, both with the use of standard locus-specific approach and of Telescope (*P*< 0.001–both; *P*= 0.011 with the standard approach and *P*= 0.001 with Telescope; 0.004 with the standard approach and *P*< 0.001 with Telescope; *P*= 0.009 with the standard approach and *P*< 0.001 with telescope, respectively) compared to ‘old’ elements (age > 20MY).

Compared to ‘intermediate age’ and ‘old’ elements, ‘young’ elements demonstrated significantly higher bias in small- and medium-length datasets (both single- and paired-end), both with the standard locus-specific and the Telescope approach, while in the PacBio-HiFi long-read simulated dataset, we observed significantly higher bias only with the use of the standard locus-specific approach and only for the ‘intermediate age’ elements (*P <*0.05 in all cases) (Figure 2). Compared to ‘intermediate age’ and ‘old’ elements, ‘young’ elements demonstrated significantly higher bias, when we used the standard locus-specific approach and the telescope analysis for standard PacBio simulated data only when it was processed with the NGMLR aligner (*P <* 0.001 and *P =* 0.001, respectively), while no significant differences in the bias were found in the Bowtie2 standard analysis (Figure 2). No statistically significant differences were found in the calculated bias between the mapped and assigned reads per locus between elements of ‘intermediate age’ and ‘old’ elements (Figure 2).

### These results are not significantly modified when HML-2 proviral elements are separately analyzed

This sensitivity analysis indicates similar results in terms of the accuracy of the detection of the expression of HML-2 elements at an mRNA level and the factors that affect it. We have shown that the locus-specific expression bias is still present with the use of the detection methods described and that Telescope implementation can partially improve the accuracy of the detection (Supplementary Table S2). Use of longer reads significantly improves the accuracy of mRNA expression, while the highest Percent Errors occurred with the use of the standard PacBio error profile, regardless of the aligner used, and the PacBio-HiFi error profile (Supplementary Table S3). Generally increased age of integration of proviral elements was found to be correlated with lower expression bias, as calculated with the use of the Percent Error (Supplementary Table S4). ‘Young’ HML-2 proviral integrations compared to ‘intermediate age’ proviral HML-2 elements demonstrated higher percent errors with the use of 76nt long reads, both with single- and paired-end layout, and 150nt long reads with single-end layout with the use of Telescope, and 150nt long reads with paired-end layout with the use of the standard locus-specific approach (*P*= 0.021, all cases). Additionally, compared to ‘old’ integrations, ‘young’ proviral integrations are characterized by higher percent errors with the use of 76nt long reads, both with single- and paired-end layout, and 150nt long reads with single-end layout with the use of Telescope (*P*= 0.021, all cases). No significant differences were identified between the expression bias of ‘intermediate age’ and ‘old’ proviral element detection with the platforms examined.

## Discussion

We have demonstrated that RNA expression analyses focusing on specific retroelement integrations is vulnerable to locus-specific expression bias with second generation and standard-error third generation high-throughput sequencing technologies, even with the use of tools specially designed to resolve transcription per integration site. We show that the main limitation, imposed by these sequencing technologies for the accurate quantification of the expression of endogenous retroviral integration at a locus-specific level, pertains mainly to the available read-lengths in technologies with low error rates (25). Conversely, most long-read high-throughput sequencing technologies entail higher error rates (13), which with the use of a standard locus-specific analysis hinder the accurate detection of HERV transcription levels, and despite the use of novel bioinformatics software especially designed to address the issue of high error rates, the result is still far from optimal. In all cases, falsely up- and downregulated HERV integrations sites are detected, which may lead to biased conclusions on the role of certain integrations, that are found erroneously differentially expressed.

Crucially, we showed that the Family-level approach yielded accurate results in the detection of HERV transcription, as the calculation of the sum of all the transcripts mapped in HERV loci can be utilized to give an accurate picture of the transcription of a particular family. Nevertheless, this approach is not without its limitations. Despite the lower bias in the evaluation of the transcription of a family in total, this approach lacks the benefit of the per site expression resolution, which is pivotal in the deciphering of HERV-mediated biological processes, where the resolution of the expression of individual loci may be of pathophysiological and therapeutic interest (26). Additionally, it is reasonable to assume that in the simultaneous presence of both up- and downregulated HERV loci within the same family, such an approach may potentially blur the landscape of the HERV expression and obscure the effects of each of the dysregulated loci.

Moreover, we show that one key factor in the successful determination of locus-specific HERV transcription, is the estimated integration age of the locus in question, as the accuracy of the determination of the locus-specific expression levels is significantly lower for younger integrations. This can be attributed to the significantly less amount of time that has elapsed since their initial integration in the human genome, which renders these elements better-preserved with more similar sequence to their progenitor virus. Conversely, older integrations have become more unique as they have accumulated more mutations during evolutionary time, as implied by their improved detection even with the use of short-read sequencing technologies. We intuitively also expect that the observed age-dependent bias increases with the number of copies of the retroelements from the same family because the number of highly similar sequences belonging to different integrations is directly dependent on the family copy number. These results were generally reproducible when the proviral elements included in this analysis were analyzed separately, despite the smaller number of observations included in our sensitivity analysis compared to the main one.

The development of strategies for the detection of HERV and other retroelement expression is a challenging field, both for the selection of the appropriate sequencing technology and the correct bioinformatics protocol. HERVs may participate in the development of human disease either through their products (7,27,28) or by interfering in the expression of human genes at a variable distance of their integrations (29). In the former case, the examination of the role of the endogenous retroviral compartment in human disease can be carried out by the isolation and quantification of HERV products, such as proteins. In the latter case, however, when the quantification of the transcription of a specific integration site is of interest, tools that enable the detection of the per locus expression often offer valuable information with regards to the HERV locus-specific transcription (11,30), even in the presence of genomic events, such as duplications of HERV-sequences, as demonstrated in this simulation analysis. Nevertheless, their results should be interpreted with caution, taking into consideration the estimated age and copy number of the elements in question.

The recognition of the expression of young integrations is more clinically relevant in the study of the role of HERVs in human pathology. In fact, younger integrations are expected to be more transcriptionally active with the ability to produce retroviral proteins, and the presence of recently integrated polymorphic sites is of interest for the disambiguation of the role of HERVs in human disease (3,31,32). Based on HML-2 LTR divergence and the high number of rare HML-2 insertional polymorphisms, recent evidence suggests that HML-2 infected and remained active in gorillas very recently (33). Thus, most notably, long-read technologies with appropriate error profiles are a promising tool that will aid the detection of rare polymorphic integrations and HERV genes with intact open reading frames, and elucidate the biology and evolution of HML-2, and HERVs in general, in humans and other primates.

Despite including one HERV family, HML-2, in the present study, we are confident that our results offer important data towards the better comprehension of the accuracy of the detection of HERV expression at an mRNA level. HML-2 has expanded in the genome of our ancestors for at least 20 million years (6,14,15). HML-2 is the last HERV to cease proliferation in the genome of our ancestors, demonstrating a wide integration age range (6), thus, it provides the ideal scenario to simultaneously study both very young HERV integrations, which are characterized by increased repetitiveness in their sequences, and also old HERV integrations, which as indicated by the evolutionary process have acquired a higher number of mutations that render these sequences more unique. Hence, utilizing this unique HML-2 feature, we are able to discern the effect of the evolutionary age of endogenous retroelements on the accuracy of their detection using multiple sequencing platforms and bioinformatics approaches for the resolution of their expression. Indeed, we demonstrate the dependence of the retroelement detection accuracy on both their inherent characteristics, i.e. their age since they stem from distinct invasion events, and on the selected sequencing platform used.

To our knowledge, this is the first study to demonstrate with the use of simulated datasets of multiple sequencing technologies and layouts, that while the Family-wide analysis, with the existing sequencing technologies despite its certain limitations, is accurate in describing the transcription of HERVs, the per integration locus approaches suffer from significant bias. Our findings have implications for mRNA studies of other multi-copy elements especially mobile elements. Most importantly, our findings underline the need for the optimization of the error profiles of the currently available long-read sequencing platforms and the importance of the age of the integrations of HERVs in the accurate quantification of their expression at a locus level. We have demonstrated a significant improvement in the accuracy of the detection of HERV transcription levels with the increasing read length in short- and medium-read technologies. Thus, the development of accurate reads longer than 750nt is highly important to enable the valid determination of the levels of HERV transcription per integration locus.

## Supplementary Material

lqae081_Supplemental_File

## Data Availability

The data underlying this article are available in the article and in its online Supplementary Material.

## Notes

Present address: Konstantina Kitsou, Department of Hygiene, Epidemiology and Medical Statistics, National and Kapodistrian University of Athens, Athens 11527, Greece.

Present address: Aris Katzourakis, Department of Zoology, University of Oxford, Oxford OX1 4BH, UK.

Present address: Gkikas Magiorkinis, Department of Hygiene, Epidemiology and Medical Statistics, National and Kapodistrian University of Athens, Athens 11527, Greece.
