# Supplementary material for: Limitations of current high-throughput sequencing technologies lead to biased expression estimates of endogenous retroviral elements
Source: NAR Genom Bioinform. 2024 Jul 9;6(3):lqae081. doi: 10.1093/nargab/lqae081 (PMC11231582; doi:10.1093/nargab/lqae081)
Supplement: lqae081_Supplemental_File [file lqae081_supplemental_file.docx]

**SUPPLEMENTARY DATA**

**Limitations of current high-throughput sequencing technologies lead to biased expression estimates of Endogenous Retroviral elements**

Konstantina Kitsou, Aris Katzourakis, Gkikas Magiorkinis*

*Corresponding Author: Gkikas Magiorkinis

Email: [gmagi@med.uoa.gr](mailto:gmagi@med.uoa.gr)

**Supplementary Table 1:**

HERV-K (HML-2) genomic coordinates in the Homo sapiens (human) genome assembly GRCh37 (hg19) that were used in this simulation analysis, their corresponding age, whether they are solitary LTRs (solo-LTRs) or proviral HML2 elements, and their categorization in this analysis. To overcome the estimated range of age of some of the integration sites, we used the mean of the minimum and maximum estimated age, as a surrogate of the actual age of the elements in question (in parentheses when applicable).

| **Supplementary Table 1: HERV-K (HML-2) genomic coordinates in the Homo sapiens (human) genome assembly GRCh37 (hg19) that were used in this work** | | | | | |
| --- | --- | --- | --- | --- | --- |
| ***Chromosome*** | ***Start*** | ***End*** | ***Estimated Age (Millions Years Ago)*** | ***Type of Element*** | ***Classification*** |
| 1 | 13678850 | 13688242 | 22.69-41.09 (mean: 31.89) | proviral | "old" |
| 1 | 15705513 | 15706472 | 6.21 | solo-LTR | "young" |
| 1 | 20356520 | 20357508 | 21.57 | solo-LTR | "old" |
| 1 | 46795434 | 46796187 | 8.83 | solo-LTR | "young" |
| 1 | 80168025 | 80168979 | 18.79 | solo-LTR | "intermediate age" |
| 1 | 100259110 | 100260111 | 16.50 | solo-LTR | "intermediate age" |
| 1 | 155596457 | 155605636 | < 2 | proviral | "young" |
| 2 | 26972732 | 26973739 | 21.50 | solo-LTR | "old" |
| 3 | 112743479 | 112752282 | < 2 | proviral | "young" |
| 3 | 196688911 | 196689921 | 25.58 | solo-LTR | "old" |
| 4 | 3980069 | 3988631 | 11.1-20.1 (mean: 15.6) | proviral | "intermediate age" |
| 4 | 122317708 | 122318709 | 20.03 | solo-LTR | "old" |
| 4 | 134967141 | 134968140 | 15.52 | solo-LTR | "intermediate age" |
| 5 | 46000159 | 46010002 | 13.39-24.24 (mean: 18.82) | proviral | "intermediate age" |
| 5 | 99941742 | 99942752 | 17.98 | solo-LTR | "intermediate age" |
| 6 | 42861409 | 42871367 | 9.83-17.81 (mean: 13.82) | proviral | "intermediate age" |
| 7 | 48029275 | 48030236 | 16.47 | solo-LTR | "intermediate age" |
| 7 | 125808213 | 125809181 | 2.54 | solo-LTR | "young" |
| 7 | 143693218 | 143694232 | 23.30 | solo-LTR | "old" |
| 8 | 7355397 | 7364859 | 4.87-8.82 (mean: 6.85) | proviral | "young" |
| 8 | 39504501 | 39505512 | 22.62 | solo-LTR | "old" |
| 8 | 47175650 | 47183661 | 23.46-42.48 (mean: 32.97) | proviral | "old" |
| 8 | 47342597 | 47343626 | 15.78 | solo-LTR | "intermediate age" |
| 11 | 62135963 | 62150563 | 19.46-35.24 (mean: 27.35) | proviral | "old" |
| 11 | 71875417 | 71876385 | 2.35 | solo-LTR | "young" |
| 11 | 118591724 | 118600883 | 13.35-24.18 (mean: 18.77) | proviral | "intermediate age" |
| 19 | 20387400 | 20397512 | 29.71-53.79 (mean: 41,75) | proviral | "old" |
| 19 | 49392892 | 49393855 | 5.57 | solo-LTR | "old" |
| 21 | 19933916 | 19941962 | 3.46-6.27 (mean: 4.87) | proviral | "young" |
| Y | 14574675 | 14575643 | 9.38 | solo-LTR | "young" |
| **Citation:** Subramanian,R.P., Wildschutte,J.H., Russo,C. and Coffin,J.M. (2011) Identification, characterization, and comparative genomic distribution of the HERV-K (HML-2) group of human endogenous retroviruses. Retrovirology, 8, 90.  *In the case of elements with an integration age <2, we used 1.9 MY as an arithmetic proxy for statistical analysis and depiction in figures.* | | | | | |

**Supplementary Data: Telescope allocates all the reads, similar to one locus, to one specific site that they appear to correspond regardless of the existence of sequences with high identity percentage.**

In our main analysis, we observed that the integration site chr1:13678850-13688242, consistently, led to low percentages of detection of the HML-2 simulated transcripts with the use of the standard locus-specific analysis, with a bias that constituted a high outlier in all the cases and this site demonstrates 100% similarity to an adjacent HML-2 provirus (located at chromosome 1, chr1:13458015-13467406), which can be attributed to a duplication event of the HML-2 provirus after its integration. The transcription of this integration site was correctly detected with the use of the Telescope software in all the cases of short- and medium-length read simulations. To investigate the allocation of the reads with Telescope, we used the paired-end layout simulated dataset of 150nt long reads as a paradigm. After extracting the sequences corresponding to the chr1:13458015-13467406 locus from the Homo sapiens (human) genome assembly GRCh37 (hg19), using the Bedtools getfasta command with default settings (16), we used the BBMap randomreads.sh command (17) to simulate the paired-end data of 150nt long reads, which were added to the initially analyzed corresponding dataset. We conducted the Telescope analysis as described in the main manuscript.

Of the 24000 pairs of reads that were simulated for both the identical loci, 23895 read pairs (99.56% of the simulated read pairs) were assigned at chr1:13458015-13467406, in this analysis. Thus, we may assume that Telescope allocates all the reads, similar to one locus, to one specific site that they appear to correspond regardless of the existence of sequences with high identity percentage to this locus.

**Supplementary Figure 1:**

The bias calculated as percent error (%) between the assigned reads and the mapped reads to each integration site correlation to the age of the HERV-K (HML-2) sequences (N=30 in each of the layouts tested). Using a standard locus-specific approach, statistically significant correlations occurred between the percent error (%) and the estimated age of the elements in all of the Illumina error profile simulated datasets (76nt single-end Illumina error-profile: p<0.001; 76nt paired-end Illumina error-profile: p=0.003; 150nt single-end Illumina error-profile: p<0.001; 150nt paired-end Illumina error-profile: p=0.003; 750nt HiFi error-profile: p=0.015). With the use of the Telescope, statistically significant correlations between the calculated Percent Error (%) and the estimated age of the elements in 76nt single-end Illumina error-profile, 76nt paired-end Illumina error-profile, 150nt single-end Illumina error-profile, 150nt paired-end Illumina error-profile datasets (p<0.001 in all cases).


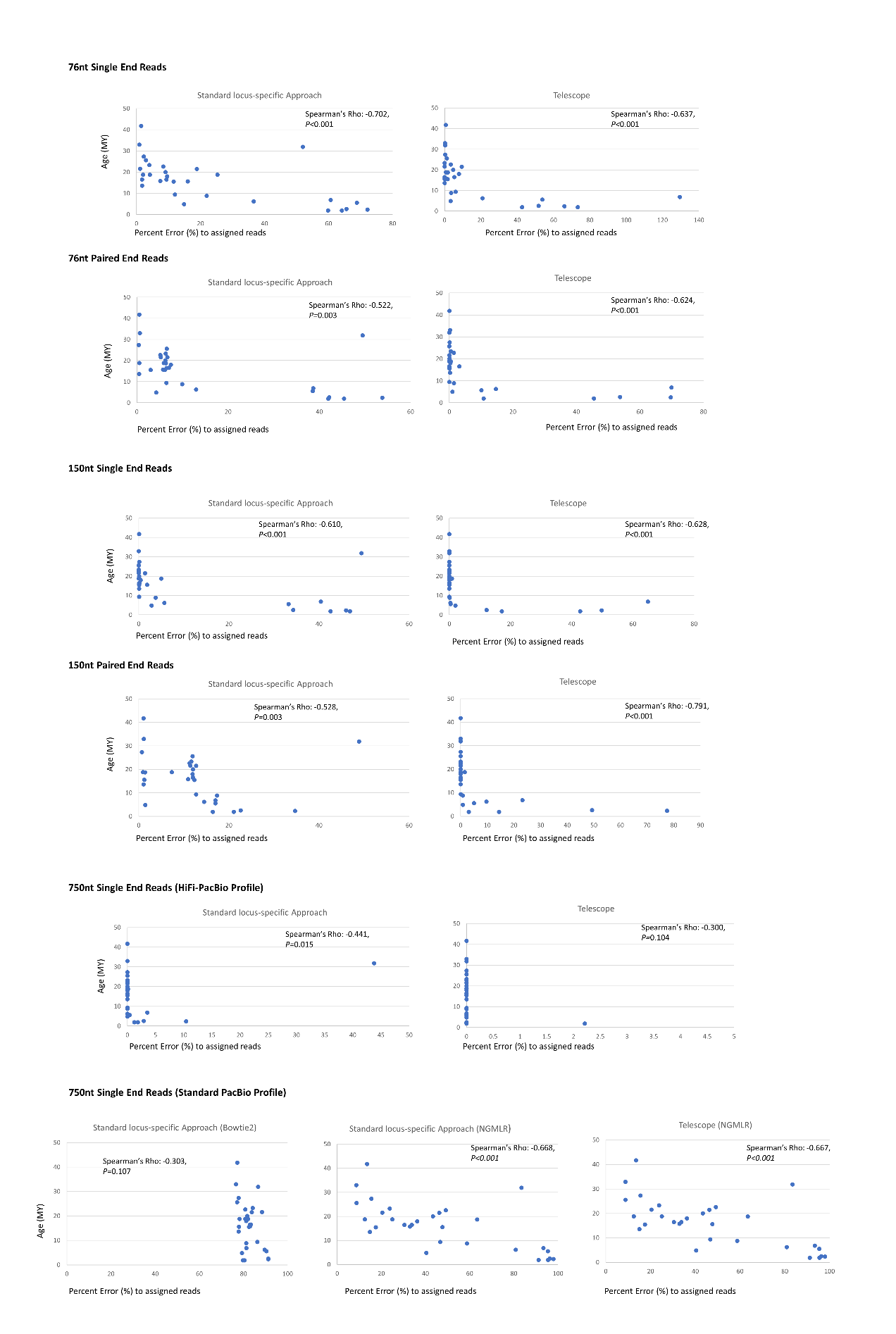


**Supplementary Table 2:**

To objectively estimate the occurring differences between the number of the assigned reads to each integration site in the simulated datasets where only reads corresponding to HERV-K (HML-2) proviral elements were included and the number of the reads that were allocated to each of the HML-2 integration sites during the analysis, with each of the approaches described above, we used the Percent Error (%), calculated as: Percent Error (%) = (|assigned-mapped|/assigned) * 100%. Percent Error (%), as a measure of the bias (difference between the assigned and the mapped reads per integration site) in simulated datasets where only proviral elements are included and comparison between the standard locus-specific approach and the use of Telescope.

| **Supplementary Table 2: Percent Error (%) in each of the simulated datasets and comparison between the two approaches used** | | | | | | | | | | |
| --- | --- | --- | --- | --- | --- | --- | --- | --- | --- | --- |
|  |  |  |  | **Locus-specific Approach** | | | **Telescope** | | |  |
| **Error Profile** | **Length (nt)** | **Aligner** | **Layout** | **median** | **minimum** | **maximum** | **median** | **minimum** | **maximun** | **p-value** |
| Illumina | 76 | Bowtie2 | single-end | 15.44 | 0.89 | 64.28 | 0.51 | 0.05 | 121.60 | *0.043* |
| Illumina | 76 | Bowtie2 | paired-end | 3.63 | 0.40 | 49.47 | 0.06 | 0.00 | 62.50 | *0.024* |
| Illumina | 150 | Bowtie2 | single-end | 2.35 | 0.01 | 49.06 | 0.01 | 0.00 | 61.04 | 0.082 |
| Illumina | 150 | Bowtie2 | paired-end | 1.33 | 0.71 | 48.87 | 0.00 | 0.00 | 20.65 | *0.007* |
| PacBio-HiFi | 750 | Bowtie2 | single-end | 0.00 | 0.00 | 43.79 | 0.00 | 0.00 | 2.21 | 0.222 |
| Standard PacBio | 750 | Bowtie2 | single-end | 78.75 | 76.63 | 86.63 | _ | _ | _ | _ |
|  |  | NGMLR | single-end | 44.00 | 8.67 | 95.50 | 44.00 | 8.79 | 95.50 | 0.931 |
| p-values italics indicate statistically significant results, Mann-Whitney U test (two-tailed) | | | | | | | | | | |

**Supplementary Table 3:**

| **Supplementary Table 3: Comparisons between the various platforms, when only proviral elements are used to simulate the compared datasets** | | | |
| --- | --- | --- | --- |
| **Analysis protocol** |  |  |  |
| ***Locus-specific*** | **Compared Platforms** | | **p-value** |
|  | Illumina, 76nt, single-end, Bowtie2 | Illumina, 150nt, single-end, Bowtie2 | 0.119 |
|  | Illumina, 76nt, single-end, Bowtie2 | PacBio-HiFi, 750nt, single-end, Bowtie2 | *0.001* |
|  | Illumina, 76nt, single-end, Bowtie2 | Standard PacBio, 750nt, single-end, Bowtie2 | *<0.001* |
|  | Illumina, 76nt, single-end, Bowtie2 | Standard PacBio, 750nt, single-end, NGMLR | 0.0941 |
|  | Illumina, 150nt, single-end, Bowtie2 | PacBio-HiFi, 750nt, single-end, Bowtie2 | *0.018* |
|  | Illumina, 150nt, single-end, Bowtie2 | Standard PacBio, 750nt, single-end, Bowtie2 | *<0.001* |
|  | Illumina, 150nt, single-end, Bowtie2 | Standard PacBio, 750nt, single-end, NGMLR | *0.007* |
|  | Standard PacBio, 750nt, single-end, Bowtie2 | Standard PacBio, 750nt, single-end, NGMLR | 0.149 |
|  | Illumina, 76nt, paired-end, Bowtie2 | Illumina, 150nt, paired-end, Bowtie2 | 0.863 |
| ***Telescope*** |  | |  |
|  | Illumina, 76nt, single-end, Bowtie2 | Illumina, 150nt, single-end, Bowtie2 | 0.105 |
|  | Illumina, 76nt, single-end, Bowtie2 | PacBio-HiFi, 750nt, single-end, Bowtie2 | *0.001* |
|  | Illumina, 76nt, single-end, Bowtie2 | Standard PacBio, 750nt, single-end, NGMLR | *0.008* |
|  | Illumina, 150nt, single-end, Bowtie2 | PacBio-HiFi, 750nt, single-end, Bowtie2 | 0.028 |
|  | Illumina, 150nt, single-end, Bowtie2 | Standard PacBio, 750nt, single-end, NGMLR | 0.002 |
|  | Illumina, 76nt, paired-end, Bowtie2 | Illumina, 150nt, paired-end, Bowtie2 | 0.232 |
| p-values italics indicate statistically significant results, Mann-Whitney U test (two-tailed) | | | |

**Supplementary Table 4:**

| **Supplementary Table 4: Correlations between the expression bias as Percent Error (%) and the age of integration of the HERV-K (HML-2) proviral elements in each of the simulated datasets and comparison between the two approaches used** | | | | | | |
| --- | --- | --- | --- | --- | --- | --- |
| **Error Profile** | **Length (nt)** | **Aligner** | **Layout** | **Analysis Protocol** | **Spearman's Rho** | **p-value** |
| Illumina | 76 | Bowtie2 | single-end | Locus-specific | -0.671 | *0.017* |
|  |  |  |  | Telescope | -0.573 | 0.051 |
| Illumina | 76 | Bowtie2 | paired-end | Locus-specific | -0.448 | 0.145 |
|  |  |  |  | Telescope | -0.732 | *0.007* |
| Illumina | 150 | Bowtie2 | single-end | Locus-specific | -0.510 | 0.090 |
|  |  |  |  | Telescope | -0.660 | *0.020* |
| Illumina | 150 | Bowtie2 | paired-end | Standard | -0.448 | 0.145 |
|  |  |  |  | Telescope | -0.632 | *0.027* |
| PacBio-HiFi | 750 | Bowtie2 | single-end | Locus-specific | -0.328 | 0.299 |
|  |  |  |  | Telescope | -0.453 | 0.139 |
| Standard PacBio | 750 | Bowtie2 | single-end | Locus-specific | -0.441 | 0.152 |
|  |  | NGMLR | single-end | Locus-specific | -0.664 | *0.018* |
|  |  |  |  | Telescope | -0.664 | *0.018* |
| p-values italics indicate statistically significant results (two-tailed) | | | | | | |
